# A subset of hypertrophic cardiomyopathy patients is predisposed to angulated septum

**DOI:** 10.1186/1532-429X-17-S1-P272

**Published:** 2015-02-03

**Authors:** Rebecca Liu, Gladys Rodriguez, Steffen Huber, Sameh Hozayen, William J McKenna, Daniel Jacoby

**Affiliations:** Internal Medicine, Yale University School of Medicine, New Haven, CT USA; Diagnostic Radiology, Yale University School of Medicine, New Haven, CT USA; University College London, London, UK

## Background

Hypertrophic cardiomyopathy (HCM) is a complex genetic disease with marked morphofunctional heterogeneity. Some HCM patients develop obstructive symptoms later in life, long after cessation of hypertrophic progression. The mechanism underlying this phenomenon is poorly understood. It is known that aorto-septal angulation progresses with age. However, the relationship between age, septal angulation, and HCM subtype has not been explored. In this present study, we examined the relationship between age, aorto-septal angulation and subtypes of HCM.

## Methods

Control normal subjects (n=19) and consecutive HCM patients with apical, concentric, and septal subtypes (n=53) were identified from the MRI database at Yale-New Haven Hospital. Angulated septal angle, the angle between the right septal surface and anterior aortic wall during end systole and diastole, was measured blindly by two readers. Disagreements between two reads >10˚ were excluded. In addition, we further age stratified our cohort of septal subtype (above or below 40 years) to explore differences in the pattern of aorto-septal angle over age.

## Results

Patients with septal, but not apical or concentric subtypes, exhibit more acute angulated septum versus controls in end-systole (p=0.008, 0.326, and 0.167, respectively). The acuity of this angle increased with age in controls (p=0.0009). Interestingly, HCM patients with septal hypertrophy deviated from this pattern, demonstrating early angulation without progression over age (p=0.918). The associations above remained significant in end-diastole.

## Conclusions

This novel finding suggests that HCM patients with septal, but not apical or concentric patterns of hypertrophy are predisposed to angulated septum regardless of age. Future studies are needed to assess correlations of symptom severity or obstruction with angle acuity. This will offer insight to the possible etiopathological link between angulated septum and morphofunctional subtype of HCM.

**Figure 1:**
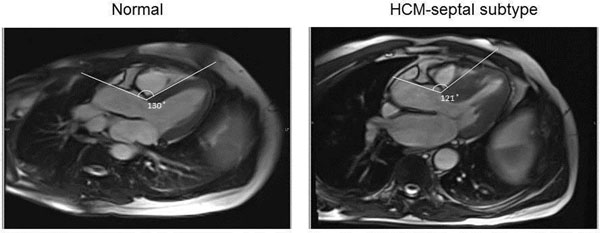
Patients with septal variant of hypertrophic cardiomyopathy exhibit more acute angulated septum than normal controls. The angulated septal angle, measured as the angle between the right septal surface and anterior aortic wall, is shown in the MRI of a normal control (left) and a patient with septal variant of HCM (right).

**Figure 2:**
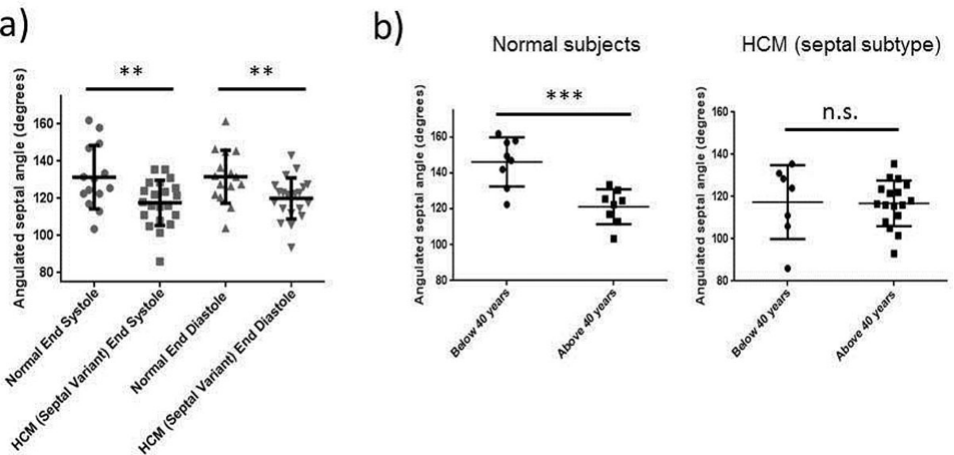
A subset of hypertrophic cardiomyopathy patients is predisposed to angulated septum. a) Patients with septal variant of HCM exhibit more acute angulated septum compared to normal controls in both end-systole and end-diastole. b) While normal subjects demonstrate progression of angulated septum over age, HCM patients with septal hypertrophy deviate from this pattern, with no progression. *** p<0.001, ** p< 0.01, n.s.= non-significant.

